# The first engkabang jantong (*Rubroshorea macrophylla*) genome survey data

**DOI:** 10.1016/j.dib.2024.111248

**Published:** 2024-12-20

**Authors:** Hung Hui Chung, Asmeralda Ai Leen Soh, Melinda Mei Lin Lau, Han Ming Gan, Siong Fong Sim, Leonard Whye Kit Lim

**Affiliations:** a Faculty of Resource Science and Technology, Universiti Malaysia Sarawak, 94300 Kota Samarahan, Sarawak, Malaysia; b Patriot Biotech Sdn Bhd, 47500 Subang Jaya, Selangor, Malaysia; c Centre for Integrative Ecology, School of Life and Environmental Sciences, Deakin University, Geelong, Victoria, Australia

**Keywords:** Engkabang jantong, *Rubroshorea macrophylla*, Genomic landscape, Microsatellite, Illumina

## Abstract

The engkabang jantong (*Rubroshorea macrophylla*) is one of the most indispensable tree species for reforestation due to its high survival rate and rapid growth rate. Due to relatively low genetic interest of this tree species, its genomic landscape has since faced scarcity, impeding our further elucidation on genes that are involved in expressing its aforementioned superior properties. In this study, we performed genome survey and microsatellite analysis of engkabang jantong. Based on the results, the estimated genome size of this species is 312,071,515 bp with 18.43 % repeated sequences and 1.16 % heterozygosity. BUSCO analysis unearthed that 83.5 % of the contigs are single-copy genes whereas 12.7 % of them are duplicated. Only 2.8 % and 1 % of them are fragmented and missing respectively. The short-read sequencing results obtained from the Illumina platform in this study will be essential to complement the Nanopore long-read sequencing results in hybrid genome assembly endeavors in the near future.

Specifications Table

**Table:** 

Subject	Biological Sciences.
Specific subject area	Genomics
Type of data	Sequencing raw reads, Table and Figure.
Data collection	The extracted engkabang jantong DNA was sheared into 350 bp fragments using Covaris Ultrasonicator. Then, library preparation was done using NEB Ultra II library preparation kit according to manufacturer's protocol. The assembled library was then subjected to sequencing via Illumina NovaSeq 6000 platform*.*
Data source location	The collection of engkabang leaves from a single individual tree is under the permission of Sarawak Forestry Corporation (Reference Number: SFC.810-4/6/1(2022)). The engkabang leaves are provided by the ranger of the Sarawak Forestry Corporation. The collection of leaves is carried out at Semenggoh Wildlife Center, Kuching, Sarawak, Malaysia (1.402258002376039, 110.31446195505569). The preserved dry engkabang leaves were deposited in UNIMAS Herbarium with accession number HB008123.
Data accessibility	The sequencing reads used in the analysis are available under the NCBI BioProject PRJNA1127791 Repository name: NCBI GenBank database Data identification number: BioProject PRJNA1127791 Direct URL to data: https://www.ncbi.nlm.nih.gov/bioproject/?term=PRJNA1127791 Instructions for accessing these data: Click on the link provided above
Related research article	None

## 1 Value of the Data

• First genome survey conducted on engkabang jantong.
• Enable for genome-wide association studies.
• Enable future research on genotypes contributing to high quality timber and fatty acids produced by this species.

## 2 Background

The engkabang jantong (*Rubroshorea macrophylla*) belongs to the family Dipterocarpaceae. It plays significant roles in the ecology system, aquaculture feed and reforestation due to its rapid growth rate and high survival rate [[1], [2], [3], [4]]. Besides its trunk that produces high quality timber, its fruit (also known as illipe nut) is highly encompassed with high quality oil and fatty acids [5] and is natural food loved by a highly priced fish species, namely the empurau in the wild [[6], [7], [8]]. Its unique fatty acid content and fragrance is what makes the empurau fish taste scrumptiously palatable with unique texture [9,10].

Due to the relatively low genetic interest placed into this tree species, the only genomic data found on *R. macrophylla* was that from the complete chloroplast genome sequencing performed by [11]. In this study, we conducted a genome survey on the engkabang jantong whole genome before performing BUSCO, k-mer and microsatellite analyses to characterize the whole genome. Furthermore, we also further characterize the genome with analyses such as functional annotation and phylogenetic tree construction. It is hoped that the short-read sequencing results obtained from the Illumina platform in this study will be essential to complement the Nanapore long-read sequencing results in hybrid genome assembly endeavors in the near future.

## 3 Data Description

**Fig. 1:**
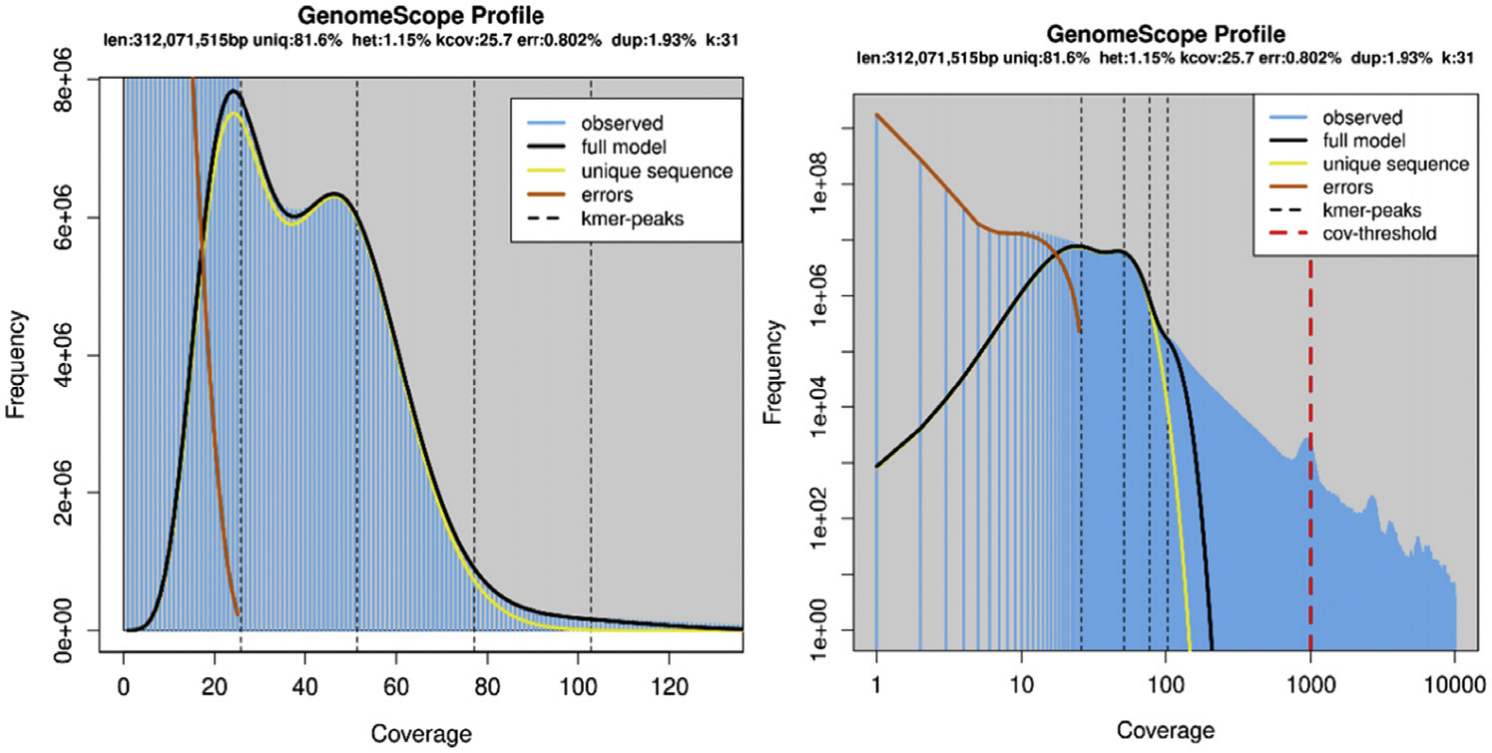
Estimation of genome size, repeat content, and heterozygosity was visualized using the GenomeScope webserver, based on k-mer 31 (read length = 150 bp; k-mer max coverage at 1000). The y-axis displayed the number of k-mers while the x-axis depicted the coverage.

The engkabang jantong genome contigs were filtered and screened directly after sequencing, unraveling a sum of 29.85 Gb of clean reads generated with ∼68.6× coverage (Fig. 1). A total of 96,606 contigs with total contig length 435,158,746 bp was reported in this study. The lengthiest contig is 799,893 bp long while the genome size was estimated at 312,071,515 bp (∼312 Mbp) (Table 1). This genome size is close to that of its genus counterparts such as *Rubroshorea robusta, Rubroshorea leprosula, Rubroshorea henryana* and *Rubroshorea roxburghii* with genome size estimated at 357.11 Mbp, 323.6 Mbp, 302.6 Mbp and 306.2 Mbp [12,13]. respectively. The engkabang jantong genome GC content documented in this study is 33.38 %, which is very similar to that of *R. roxburghii* (33.2 %) [12], *R. leprosula* (33.4 %) [12], and *R. robusta* (33.69 %) [13]. This demonstrated the high conservation of GC content and genome size across the *Rubroshorea* genus. BUSCO analysis unearthed that 83.5 % of the engkabang jantong genome contigs are complete and single-copy protein-coding genes, whereas 12.7 % of them are complete and duplicated genes. Only 2.8 % and 1 % of them are fragmented and missing respectively, according to the BUSCO analysis in this study. The heterozygosity of the engkabang jantong recorded in this study is ∼1.16 %, which is considered high when compared to other plant species like Satsuma (0.435 %), sago palm (0.63 %), pummelo (0.022 %), date palm (0.46 %), sweet orange (0.716 %) and Clementine (0.462 %) [14]. The repeated sequence percentage of the engkabang jantong genome is 18.43%, which is very much lower compared to that of its genus counterpart, *R. leprosula* (33 %) (Ng et al., 2021) as well as other plant species, for instance, *Oryza sativa* (39.5 %), oil palm (57 %), sago palm (35.7 %), *Vitis vinifera* (41.4 %), date palm (38.41 %) and *Populus trichocarpa* (42 %) [14]. The heterozygosity ratio and repeated sequence percentage are responsible for the fabrication and splicing in the genome, they also reflect the heterogeneity of the plant habitat, contributing to its biodiversity [15].

**Table 1:** The genomic data summary of the engkabang jantong genome.

Organism	*Rubroshorea macrophylla*
Bioproject	PRJNA1127791
Biosample	SAMN42019830
Sequence Read Archive (SRA)	SRS21753026
Complete and singe copy BUSCOSs	83.5 % (355)
Complete and duplicated BUSCOs	12.7 % (54)
Fragmented BUSCOs	2.8 % (12)
Missing BUSCOs	1.0 % (4)
Scaffold N50	22 kb
Contig N50	15 kb
Number of scaffolds	70,981
Number of contigs	96,606
Genome heterozygosity	1.16 %
Genome repetitiveness	18.43 %
Predicted genome size	312,071,515 bp
GC content (%)	33.38 %

**Fig. 2:**
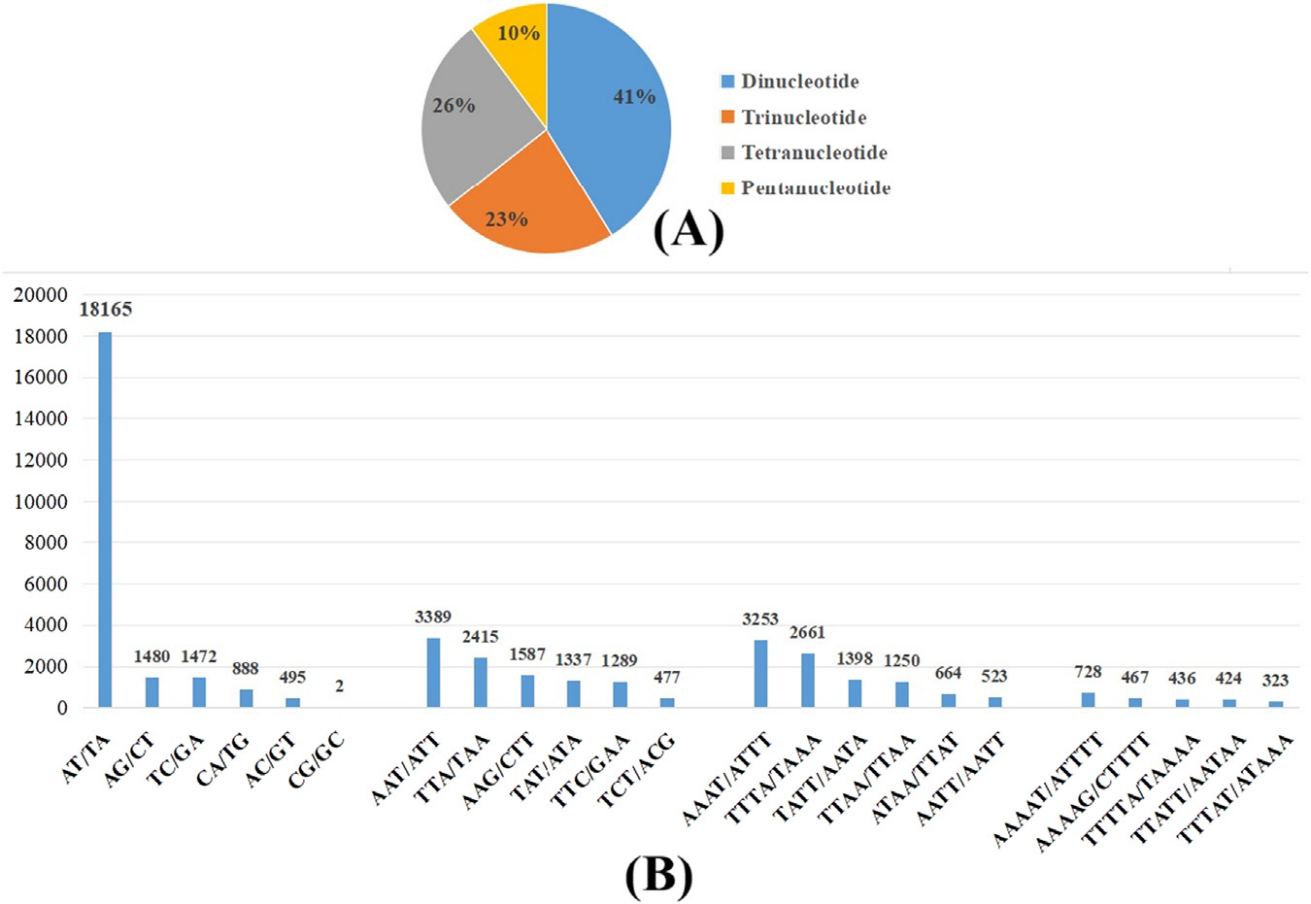
(A) The summary of microsatellite populations in the engkabang jantong genome. (B) The top six highly abundant microsatellite species for each microsatellite population in the engkabang jantong genome.

To date, there is no genome wide microsatellite analysis done onto any Dipterocarpaceae family members. The genome-wide microsatellite analysis conducted in this study unearthed that a sum of 54,607 short sequence repeats (SSRs) were discovered within the engkabang jantong genome. The dinucleotide repeats cover majority (22,502, 41 %) of the short sequence repeats identified in this study with minimum four repeats (Fig. 2A). The least found short sequence repeats are the pentanucleotide repeats (5584, 10 %). A similar phenomenon was seen in sago palm genome whereby the most abundant SSRs is the dinucleotide repeats (62.24 %) whereas the least discovered SSRs is the pentanucleotide repeats (5.91 %) [14]. The most abundant SSR species found is the AT/TA with 18,165 (33.26 %) found within the engkabang jantong genome, which made them the top among the dinucleotide repeats identified in this study (Fig. 2B). The top three trinucleotide microsatellites of the engkabang jantong genome are AAT/ATT (6.21%), TTA/TAA (4.42 %) as well as TAT/ATA (2.91 %). The AAAT/ATTT, TTTA/TAAA, and TATT/AATA topped the engkabang jantong genome tetranucleotide SSRs chart with compositions of 5.96 %, 4.87 % and 2.56 % correspondingly. The most abundant engkabang jantong genome pentanucleotide microsatellite is the AAAAT/ATTTT (1.33 %), followed by AAAAG/CTTTT (0.86 %) and TTTTA/TAAAA (0.8 %). Interestingly, the top three engkabang jantong genome tetranucleotide and pentanucleotide microsatellites mirrored that of the sago palm genome with differing population sizes [14]. These microsatellite data generated in this study lay imperative groundwork for the DNA fingerprinting and barcoding endeavor for species identification across the Dipterocarpaceae family members in the future.

**Fig. 3:**
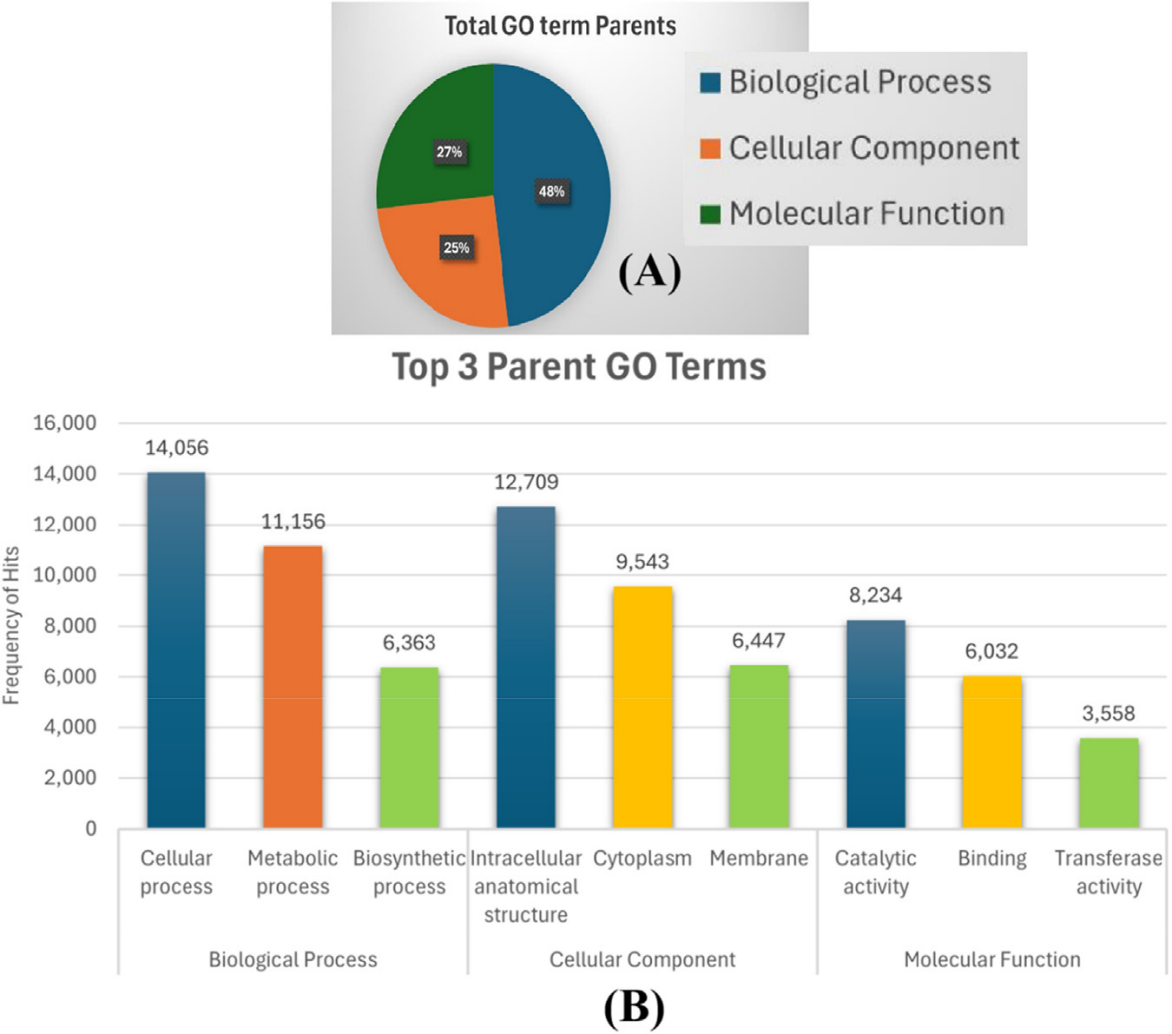
(A) The summary of parent GO terms found within the engkabang jantong genome. (B) The top three GO IDs for each of the parent GO terms identified in the engkabang jantong genome.

The functional annotation of Gene Ontology (GO) terms using EggNOG mapper v2 [16] revealed 41,061 genes associated with GO term IDs within the engkabang jantong genome. Upon further functional annotation using TbTools II [17], 18,173 genes were found to have link with 94 parent GO terms. Almost half (48%) of the identified genes are associated with the biological process parent GO term. A quarter of them (25 %) are grouped under cellular component parent GO term while the remaining (27 %) are housed under the molecular function parent GO term (Fig. 3A). Zooming into each parent GO terms identified in the engkabang jantong genome in this study (Fig. 3B), we depicted the top three hits for each parent GO term. Under the biological process parent GO term, the cellular process topped the chart with 14,056 hits, followed by metabolic process (11,156 hits) as well as biosynthetic process (6363 hits). Under the cellular component parent GO term, the intracellular anatomical structure is the leading hit with 12,709 hits, the second leading hit is the cytoplasm (9543 hits) and the third one is the membrane (6447 hits) within the engkabang jantong genome. Under the engkabang jantong genome molecular function parent GO term, the catalytic activity is the most frequently found hit with 8234 hits, while the second and third ones are binding (6032) and transferase activity (3558) respectively. The abundant metabolic and biosynthetic genes found within the engkabang jantong genome is postulated to have associated with its capability to produce huge amount of high quality cell wall, secondary cell wall (timber), fatty acids (fragrant oleoresin) as well as for defense mechanism [12].

**Fig. 4:**
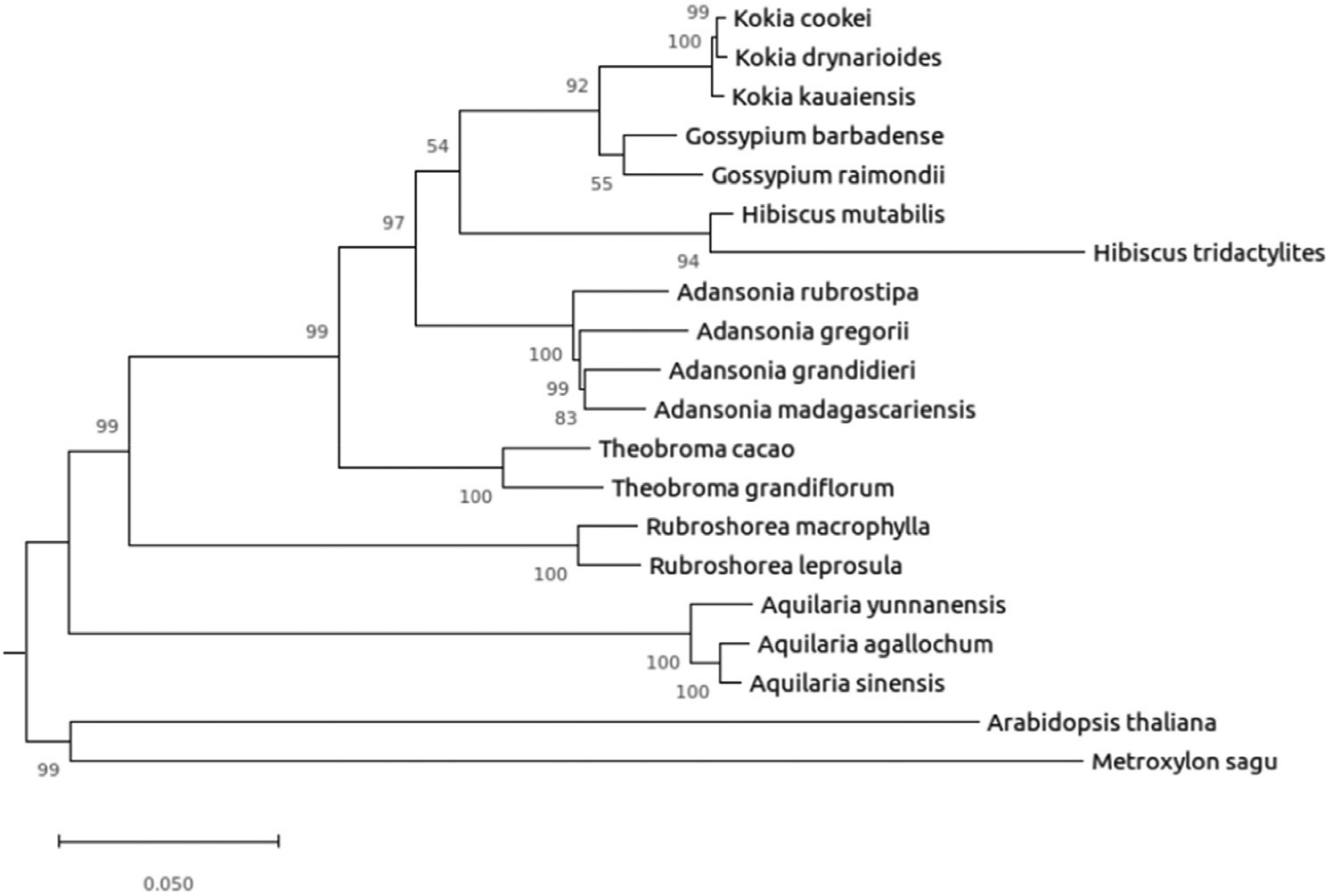
The phylogenetic tree constructed across 18 plant species with two outgroups based on 1000 bootstrap replications.

A phylogenetic tree was constructed to include a sum of 18 plant species with two outgroups (*M. sagu* and *A. thaliana*) with 1000 bootstrap replications (Fig. 4). All the 18 plant species form monophyletic clade with their respective genus counterparts with varying bootstrap values from 55 to 100. The engkabang jantong formed a strong clade with its genus counterpart, *R. leprosula* with the maximum bootstrap value of 100. This similar phenomenon was seen in other phylogenetic tree constructed such as the sago palm [14] whereby the genus counterparts shared the same clade with maximum bootstrap values. This is essential for accurate and precise species identification and also beneficial for the discovery of hybrid species in the future if there is any occurrence happening in the future. The results from this study also offer the engkabang jantong genome as a reference genome for future genome sequencing and assembly of other currently yet to be sequenced Dipterocarpaceae family members genomes.

## 4 Experimental Design, Materials and Methods

### 4.1 Sampling, DNA extraction and genome sequencing

The leaf tissues of *Rubroshorea macrophylla* were obtained from Semenggoh Wildlife Centre (1°23′59″N 110°19′27″E) with authorization from the Sarawak Forestry Corporation (Reference Number: SFC.810-4/6/1(2022)). The DNA extraction was done emulating that from [18]. The DNA extracted was quality checked using DeNovix DS-11+ spectrophotometer (DeNovix, USA) and agarose gel electrophoresis analysis. The extracted engkabang jantong DNA was sheared into 350 bp fragments using Covaris Ultrasonicator (Covaris, United Kingdom). Then, library preparation was done using NEB Ultra II library preparation kit (NEB, United Kingdom) according to manufacturer's protocol. The assembled library was then subjected to sequencing via Illumina NovaSeq 6000 platform. Adapter- and quality-trimming was performed using fastp v. 0.18 onto the raw pair-end reads.

### 4.2 Genome completeness, functional annotation, microsatellite and phylogenetic analysis

BUSCO V5.7.1 [19] was employed to assess the genome completeness of the engkabang jantong genome. Various genomic matrices were examined utilizing QUAST v5.2.0 [20]. The k-mer frequency distribution data was generated using Jellyfish v2.3.0 [21] at k-mer size of 31. GenomeScope webserver [22] was used for the visualization of various genomic matrices based on parameters, namely read length 150 bp and K-mer coverage of 1000. AUGUSTUS v3.4.0 [23] was utilized to predict the protein-coding genes from the engkabang jantong whole genome with reference to pre-trained model species *Theobroma cacao*. Functional annotation of all the predicted protein sequences was conducted using EggNOG mapper v2 [16] on the eggNOG 5 database, with E-value set at 0.001. These genes were further functionally annotated using TbTools II [17]. Microsatellite analysis was also performed using Kmer-SSR [24]. Only microsatelllites with more than four repeats were selected for further analysis. A total of 20 plant species was selected for phylogenetic tree construction with *Metroxylon sagu* and *Arabidopsis thaliana* as outgroups. The ‘complete and single-copy’ protein sequences of the 20 plant species obtained from BUSCO analysis were subjected to multiple sequence alignment using MAFFT v7.471 [25]. MEGA 11 [26] was employed to construct Neighbor-Joining (NJ) tree with 1000 bootstrap replications.

## Limitations

The limitation of this study is that the Illumina sequencing platform only generates short reads. The number of complete and single copy BUSCO of 83.5 %, this number can be further increased with long-read Nanopore sequencing platform and then subsequently conducting a hybrid genome assembly [27].

## Ethics Statement

The authors have read and follow the ethical requirements for publication in Data in Brief and confirming that the current work does not involve human subjects, animal experiments, or any data collected from social media platforms.

## CRediT authorship contribution statement

**Hung Hui Chung:** Conceptualization, Funding acquisition, Writing – review & editing. **Asmeralda Ai Leen Soh:** Data curation, Writing – original draft. **Melinda Mei Lin Lau:** Data curation. **Han Ming Gan:** Methodology, Conceptualization, Writing – review & editing. **Siong Fong Sim:** Writing – review & editing. **Leonard Whye Kit Lim:** Writing – original draft, Writing – review & editing.

## Data Availability

NCBI GenBankRubroshorea macrophylla Genome sequencing (Original data).
